# Recent trends of biotechnological production of polyhydroxyalkanoates from C1 carbon sources

**DOI:** 10.3389/fbioe.2022.907500

**Published:** 2023-01-06

**Authors:** Subhasree Ray, Jun-O Jin, Inho Choi, Myunghee Kim

**Affiliations:** ^1^ Research Institute of Cell Culture, Yeungnam University, Gyeongsan, South Korea; ^2^ Department of Life Science, School of Basic Science and Research, Sharda University, Greater Noida, India; ^3^ Department of Medical Biotechnology, Yeungnam University, Gyeongsan, South Korea; ^4^ Department of Food Science and Technology, Yeungnam University, Gyeongsan, South Korea

**Keywords:** greenhouse gas, PHA (polyhydroxyalkanoates), methanotroph, formate, hydrogen oxidizing bacteria

## Abstract

Growing concerns over the use of limited fossil fuels and their negative impacts on the ecological niches have facilitated the exploration of alternative routes. The use of conventional plastic material also negatively impacts the environment. One such green alternative is polyhydroxyalkanoates, which are biodegradable, biocompatible, and environmentally friendly. Recently, researchers have focused on the utilization of waste gases particularly those belonging to C1 sources derived directly from industries and anthropogenic activities, such as carbon dioxide, methane, and methanol as the substrate for polyhydroxyalkanoates production. Consequently, several microorganisms have been exploited to utilize waste gases for their growth and biopolymer accumulation. Methylotrophs such as *Methylobacterium organophilum* produced highest amount of PHA up to 88% using CH_4_ as the sole carbon source and 52–56% with CH_3_OH. On the other hand *Cupriavidus necator*, produced 71–81% of PHA by utilizing CO and CO_2_ as a substrate. The present review shows the potential of waste gas valorization as a promising solution for the sustainable production of polyhydroxyalkanoates. Key bottlenecks towards the usage of gaseous substrates obstructing their realization on a large scale and the possible technological solutions were also highlighted. Several strategies for PHA production using C1 gases through fermentation and metabolic engineering approaches are discussed. Microbes such as autotrophs, acetogens, and methanotrophs can produce PHA from CO_2_, CO, and CH_4_. Therefore, this article presents a vision of C1 gas into bioplastics are prospective strategies with promising potential application, and aspects related to the sustainability of the system.

## Graphical Abstract

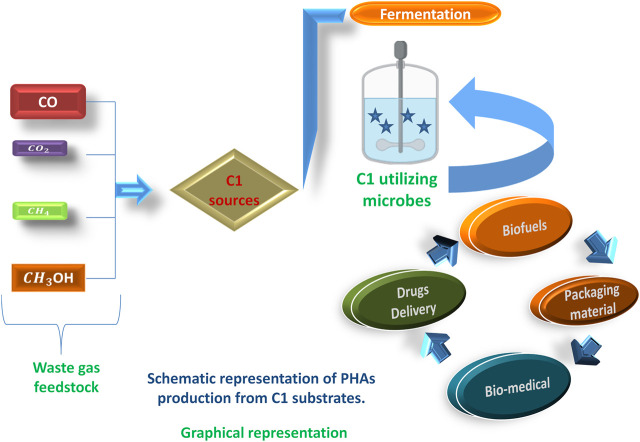

## 1 Introduction

**FIGURE 1:**
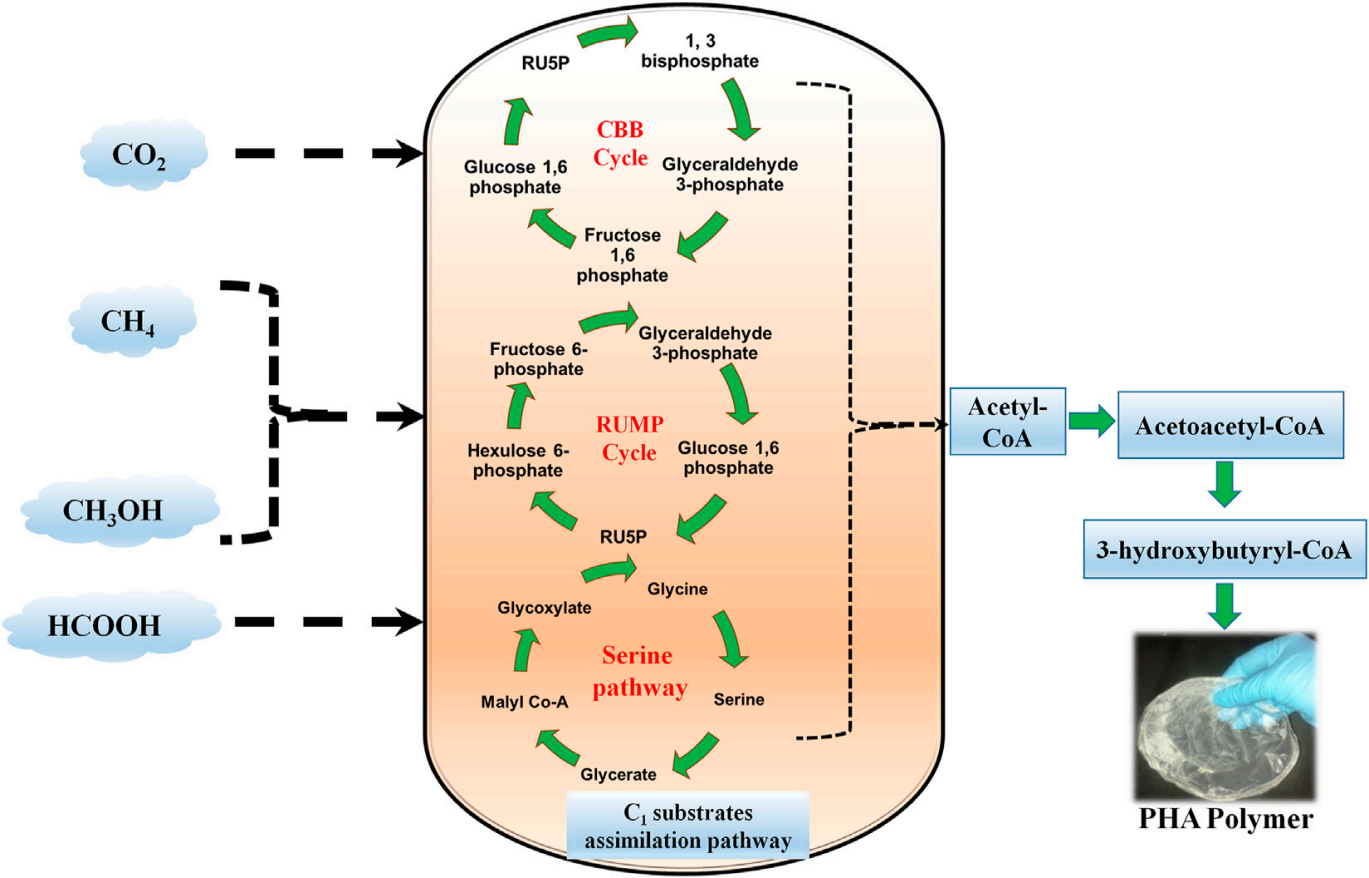
Schematic diagram showing a brief represenation of overall C1 substrates assimilation pathway for PHA production. CBS: Calvin-Benson-Bassham cycle; RUMP: Ribulose Monophosphate Pathway.

Industries and societies are facing problems with the sustainable production of value-added chemicals while seeking reductions in greenhouse gas emissions (Ai-Yaeeshi et al., 2020). Currently, fossil fuels fulfill the need for energy and chemicals and play a major role in the global economy (Abe et al., 2019). Such resources are present in limited quantities, and their excessive use lead to an environmental pollution (Ai-Yaeeshi et al., 2020). In addition to anthropogenic activities, industries are also emitting waste gases, including carbon dioxide (CO_2_), methane (CH_4_), and carbon monoxide (CO) into the atmosphere every year (Choi et al., 2020a). Several approaches, such as physical, chemical methods, are employed to reduce pollution however; physio-chemical methods require intensive energy. To overcome this problem, these waste gases (C1) can be valorized to produce value-added chemicals by biological approaches (Dürre and Eikmanns, 2015). Some microbes can grow on these C1 compounds, including methanol (CH_3_OH). Therefore, they would help to mitigate waste gases from the environment while simultaneously exploiting them to produce value-added bioproducts such as polyhydroxyalkanoates (PHAs) a biodegradable biopolymer (Dürre and Eikmanns, 2015) (Figure 1). Once realized on a large scale, this could be a paradigm approach towards the valorization of waste gases to biopolymers. Such a waste biorefinery strategy offers a great potential towards a sustainable economy (Kumar et al., 2016). PHA production from C1 carbon sources have more advances such as i) direct bioconversion process ii) fermentation approach without sterilization.

Synthetic polymers are irreplaceable materials in our daily life and are commonly used in packaging, electronic devices, and the transportation of household materials. Globally, annual plastic production has reached 311 million tons and by 2050, it is anticipated to reach up to 500 million metric tons (Liu et al., 2020). The overuse of synthetic plastics leads to a prodigious amount of waste generated in the environment. The primary disadvantage is its non-biodegradable nature and accumulation in the environment in enormous quantities. When exposed to solar radiation, synthetic plastics generate greenhouse gases such as CH_4_ and ethylene. This scenario has encouraged many researchers to search for alternative polymers. Green alternatives by replacing synthetic plastics with biodegradable polymers such as PHAs are considered sustainable approaches (Raza et al., 2018). Microorganisms accumulate PHAs as carbon reservoirs under nutrient limiting conditions (Ray and Kalia, 2017a,b; Kumar et al., 2015). They have attractive physio-chemical properties such as elastomeric, piezoelectric, biodegradability, non-toxicity, and biocompatibility (Steinbüchel and Lutke-Eversloh, 2003; Chen and Wu, 2005; Bugnicourt et al., 2014; Ray and Kalia, 2017c). The physical properties of PHA depend on its monomeric composition, which is classified according to the number of carbon chain lengths such as short-chain length (scl, C_2_-C_5_), medium-chain length (mcl, C_6_-C_14_), and long-chain length in the monomer (lcl, C_15_-C_20_) (Ray and Kalia, 2017c). To date, one hundred sixty monomers of PHAs have been identified. Depending on the monomeric composition, PHAs have various applications (Singh et al., 2015; Choi et al., 2020b). Several homopolymers and co-polymers of PHAs are used in industrial and biomedical applications such as packaging, food additives, films, fibers, drug delivery, medical implants, scaffold preparation, tissue engineering, memory enhancers, and biofuels (Ray et al., 2018). However, higher substrate cost is the limiting factor for large-scale production.

Biowastes have been a key player in the production of value added bio-products, but the availability, difficulty in pretreatment has become an issue (Kumar et al., 2013). On the other hand, anthropogenic activities including industries lead to the emission of waste gasses, which cause environmental pollution. To reduce pollution, some gas-fermenting microbes can grow on C_1_ compounds to produce PHAs and, lead to the elimination of the consumption of biowastes (Dürre and Eikmanns, 2015). Several Gram-negative bacteria are capable of producing PHAs such as *Azotobacter*, *Comamonas*, *Pseudomonas*, *Ralstonia, Cupriavidus*, *Haloferax,* and *Klebsiella*, while some Gram-positive bacteria accumulate PHAs such as *Streptomyces, Rhodococcus, Clostridium, Staphylococcus, Nocardia, Microlunatus,* and *Bacillus* from diverse substrates (Kumar et al., 2016; Ray and Kalia, 2017a).

C1 gases play a major role in global warming. Commonly, C1 gases can be generated from household wastes, industrial wastes and agricultural wastes. Thus, they can be exploited by microbes for the production of valuable bio-products such as PHAs. This approach could help to alleviate the environmental crises of global warming and plastic waste. In this present review, recent advances in PHA production approaches from carbon dioxide (CO_2_), methane (CH_4_), methanol (CH_3_OH), carbon monoxide (CO), and formate are discussed. Utilization of C1 gases by autotrophs, acetogens and methanotrophs are discussed briefly. Several approaches to enhance PHA production such as metabolic engineering, process engineering etc. are discussed. These approaches help to establish a complete closed-loop PHA production from C1 gases and its effective degradation. This review articles encompasses the utility of PHA produced by C1 sources in various biotechnological applications.

## 2 PHA production from CO_2_ by cyanobacteria

Since the industrial revolution, CO_2_, a strong greenhouse gas emitter which has reached up to 43%, and by the year 2100, it is expected to increase by 60% (Kumar et al., 2016). The microbial approach towards CO_2_ sequestration and fixation are sustainable strategies for CO_2_ mitigation and are more energy-efficient methods as compared to catalytic conversion (Claassens, 2017). Ribulose-1,5-bisphosphate and carbonic anhydrase help microbes to capture and store CO_2_ thereby reducing greenhouse gases in the atmosphere (Kumar et al., 2016). Hydrogen-oxidizing bacteria, cyanobacteria, and algae are capable of consuming CO_2_ from industrial flue gas as the sole energy source or in the form of sodium bicarbonate (NaHCO_3_) or sodium carbonate (NaCO_3_). They produce several valuable bioproducts including PHA and also treat wastewater by removing organic matter, and heavy metals (Oswald et al., 1957; Rosgaard et al., 2012; Nozzi et al., 2013; Lee et al., 2015).

**FIGURE 2:**
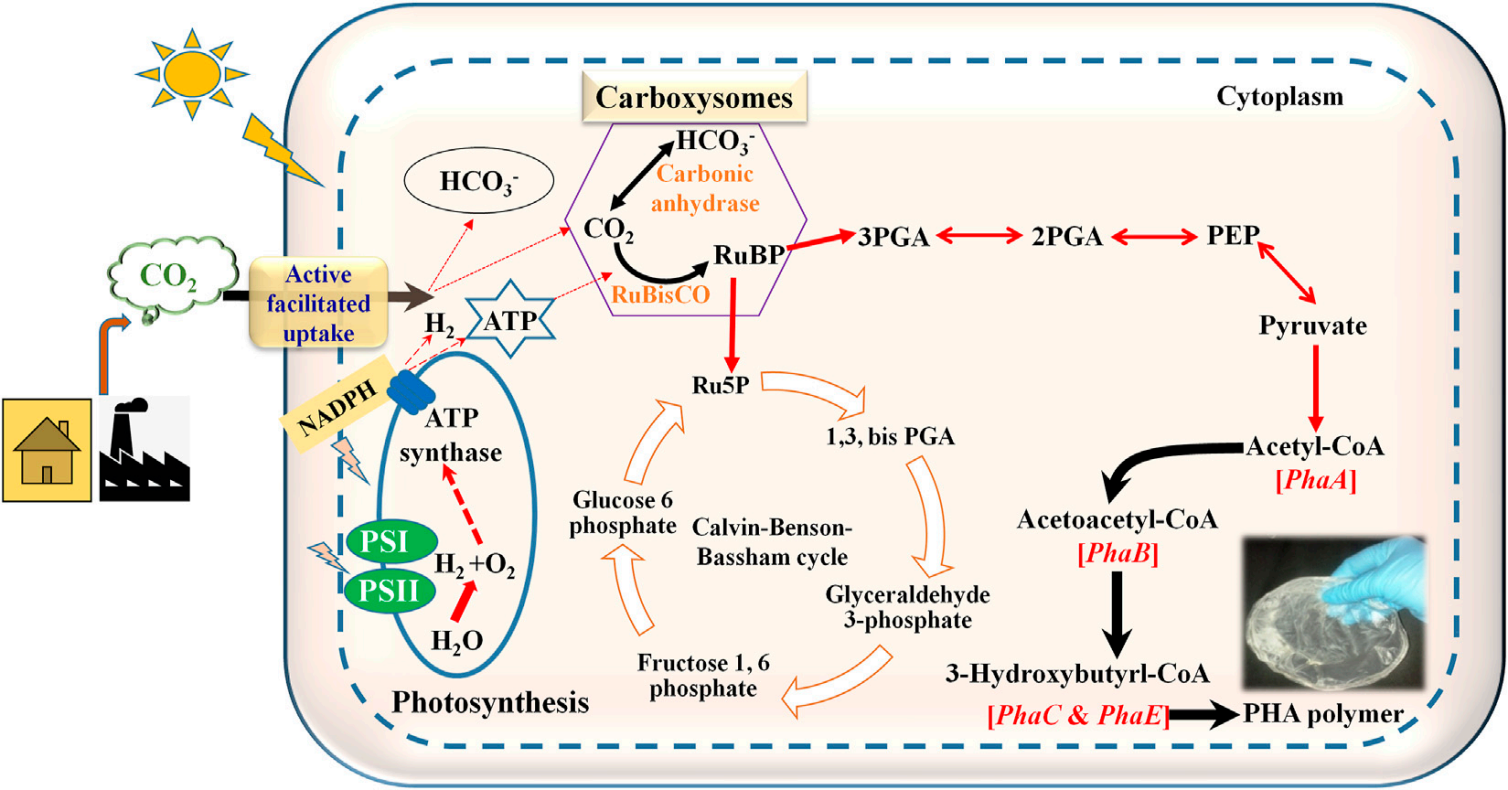
Schematic diagram showing PHA production pathway in cyanobacteria utilizing CO_2_ as carbon source. RuBP: Ribulose I, 5 his phosphate; 3PGA: 3-phosphoglyceric acid; 2PGA: 2-phosphoglyceric acid; PEP: Phosphoenolpyruvate.

**FIGURE 3:**
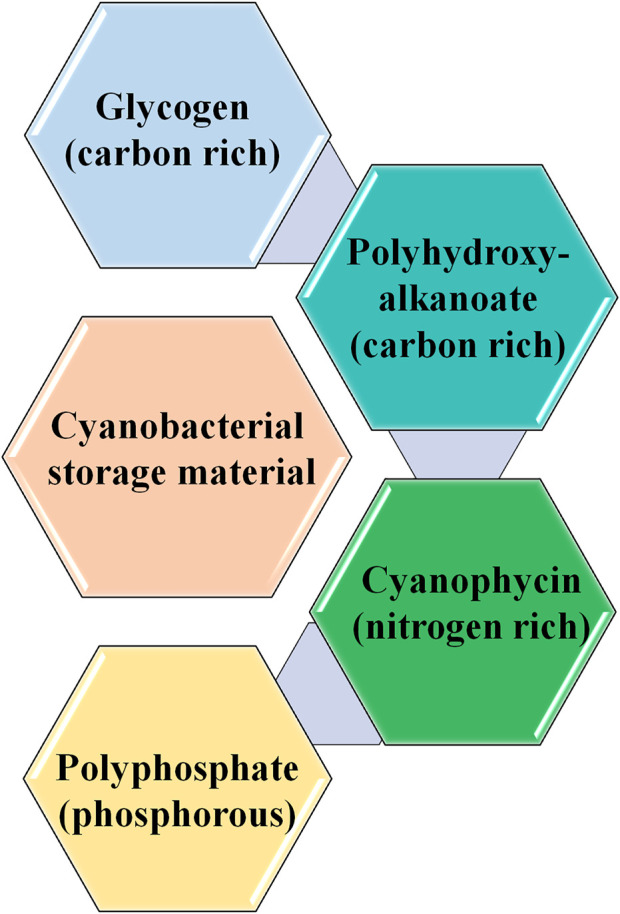
Storage materials present in cyanobacteria. The figure is a representation of various storage materials produced by cyanobacteria under nutrient limiting conditions.

A technological approach based on the cyanobacterial treatment of wastewater was successfully employed for treating municipal and industrial wastewaters (Arias et al., 2020). *Chlorella vulgaris*, *Chlorella pyrenoidosa, Rhodovulum viride, Scenedesmus obliquus,* and *Thermosynechococcus elongatus* can tolerate high CO_2_ concentrations. Cyanobacterial CO_2_ fixation is mainly influenced by physical parameters, such as type of cultivation, type of bioreactors, pH, nutrients, temperature, and light (Salehizadeh et al., 2020). However, under nutrient-limiting conditions, cyanobacteria produce different types of storage compounds, such as glycogen, polyhydroxybutyrate (PHB) (carbon-rich), cyanophycin (nitrogen-rich), and polyphosphate (Price et al., 2020) (Figure 2 and Figure 3).

Through the oxidative pentose pathway, glycogen is synthesized in the presence of sunlight and CO_2_ (Shinde et al., 2020). When compared to glycogen, PHAs could serve as long-term storage material within the cell. *Chlorogloea fritschii* was the first reported cyanobacteria to produce PHA by utilizing acetate in a photoautotrophic environment (Carr, 1966; Singh et al., 2017). Fatty acid biosynthesis and nitrogen assimilation are proposed biosynthetic pathways for the production of PHAs (Mozejko-Ciesielska et al., 2018; Price et al., 2020). Cyanobacteria are capable of performing oxygenic photosynthesis *via* Calvin-Benson-Bassham (CBB) cycle (Figure 2). They produce ATP and NADPH by capturing the thylakoid membrane from sunlight. These two intermediates are utilized for essential nutrient assimilation. The CBB pathway contains 3 phases: carboxylation, reduction, and regeneration. In carboxylation, 6 molecules of 3-phosphoglycerate (PGA) were produced by combining by 3 molecules of CO_2_ and 6 molecules of ribulose 1, 4-bisphosphate (RuBP). Subsequently, the production of NADPH and ATP occurs to reduce PGA to triose phosphate and dihydroxyacetone phosphate (DHAP). 5 molecules of PGA are utilized to reproduce 3 molecules of RuBP. Cyanobacteria uptakes Ci, CO_2_, and HCO_3_^−^ inside the cell by CO_2_ concentrating mechanism. There are five types of mechanisms; 3 for the uptake of HCO_3_^−^ (BicA, SbtA, and BCT1) and 2 for the uptake of CO_2_ (NDH-I_3_ and NDH-I_4_) in cyanobacteria. CO_2_ transport occurs through Ci transporters at the plasma membrane. Bicarbonate transporters help to transport intracellular HCO_3_^−^ ions across the plasma membrane, chloroplast, and periplasmic carbonic anhydrase convert HCO_3_^−^ to CO_2_ (Durall and Lindblad, 2015).

Furthermore, the remaining 3-PGA is utilized for the synthesis of cellular material depending upon nutrient availability. RuBisCO, phosphoribulokinase, and sedoheptulose bisphosphatase are the major enzymes of the Calvin-Benson-Bassham cycle (Kumar et al., 2018). Under nutrient-depleted conditions, 3-PGA diverts the pathway towards PHA biosynthesis (Figure 2). Several cyanobacterial species*,* such as *Spirulina maxima, Aphanocapsa* sp, *Synechocystis* sp*.* UNIWG*, Synechocystis* sp*.* PCC 6803, *Nostoc muscorum,* and *S. platensis* accumulate PHAs (Singh and Mallick, 2017).

The synthesis of PHA governed by the following enzymes:

a) ß-ketothiolase (encoded by *phaA*) catalyzes the conversion of acetyl CoA to acetoacetyl-CoA, b) acetoacetyl-CoA reductase (encoded by *phaB*) help to reduces acetoacetyl-CoA to 3-hydroxybutyryl-CoA, and c) polymerization of 3-hydroxybutyryl-CoA to PHB carried out by PHA synthase (encoded by *phaEC*) (Taroncher-Oldenburg et al., 2000; Carpine et al., 2017). PHA synthase can also incorporate other hydroxy acid monomers within the PHA. PHA synthase is categorized into four groups: 1) Class I *PhaC* subunit contains 60–73 kDa in *Ralstonia eutropha*, 2) Class II *PhaC* subunit contains 60–65 kDa in *Pseudomonas oleovorans*, 3) Class III *PhaC* and *PhaE* subunit contains 40 kDa each in *Allochromaticum vinosum, and Thiocapsa pfennigii*, and 4) *Bacillus megaterium* contains 40 kDa and 22 kDa of Class IV (*PhaC* and *PhaR*) (Ray and Kalia, 2017b). Only Class III PHA synthase has been observed in cyanobacterial species (Singh and Mallick, 2017). Cyanobacterial gene locations are slightly different from other bacteria. In bacteria, all four genes are present in one single operon, but in cyanobacteria, two separate operons are present. *PhaA* and *PhaB* are putatively co-expressed and present in the first loci while *PhaEC*, present in the second loci (Price et al., 2020).

PHB production from CO_2_ is gaining interest because of its low cost of production (Carpine et al., 2017). Under nitrogen-limiting conditions, *Synechocystis* PCC6803 produced 4.1% PHB of the total cell dry weight (Wu et al., 2001). *Synechococcus* MA19, a thermophilic organism, was reported to produce 55% PHB (Nishioka et al., 2001). However, non-thermophilic cyanobacterial species produced PHB up to 20–25% (Troschl et al., 2017a; Troschl et al., 2017b) (Table 1). However, some bacterial species such as *Cupriavidus eutrophus* B-10646, *Bacillus cereus* SS105 produced 55–85% of PHA in a batch and continuous stirred tank reactor (Table 1). Supplementation of CO_2_ to *R. eutropha* B5786 led to the production of 51% of PHA in a batch fermentation system (Table 1). Some cyanobacteria produce PHB through nitrogen fixation. *Calothrix* species produced PHB from 17 to 25% of total cell dry mass through nitrogen fixation (Price et al., 2020). *Arthrospira subsalsa* is a species of filamentous cyanobacteria that produces up to 14.7% PHB under increased salinity (Shrivastav et al., 2010). Acetate addition to the system led to the enhancement, in PHB content up to 40% by another group of cyanobacteria *Nostoc*. Further addition of propionate and valerate to the CO_2_-containing medium resulted in an enhanced P (3HB-co-3HV) content (Bhati Malick, 2015). By contrast, under phosphorous limiting conditions, co-utilization of acetate and fructose enhanced the PHB production up to 38% (w/w) of cell dry mass of *Synechocyctis* PCC6803 (Panda and Malick, 2007). Other cyanobacteria, such as *Calothrix scytonemicola* TISTR 8095 produced PHB up to 356.6 mg/L in 44 days under nitrogen limitation (Kaewbai-ngam et al., 2016). Microbial mutualism have the potential role in the bioproduction of value-added byproducts. In one study, *Azotobacter vinelandii,* a diazotroph and *S. elongates* PCC7942 co-cultured for the production of PHB by fixing CO_2_ from the atmosphere. Here, ^13^C bicarbonate was added to increase the cell growth and produced ^13^C labeled PHB. This study proved the model for cross feeding between two organisms (Smith Francis, 2016). Co-cultures containing *S. elongatus cscB* and *P. putida cscAB* utilized CO_2_ for the production of PHA in two-phase systems. In the first phase, *S. elongates cscB* fixes and converts CO_2_ to sucrose *via* the Calvin-Benson-Bassham cycle pathway. Sucrose will then release into the culture supernatant through heterologous sucrose permease *cscB* activity. *P. putida cscAB* used sucrose as a carbon source to produced PHA up to 23.8 mg/L/h with a yield of 156 mg/L after 16 days. Here, 3-hydroxydecanoic acid was found to be the major monomer (Löwe et al., 2017). The first report of mcl-PHA production was found in co-cultures containing *S. elongates* and *P. putida.* Here *P. putida* was utilized as chassis for the generation of PHA by operating metabolic pathways (Löwe et al., 2017). Some photosynthetic bacteria utilize CO_2_ to produce PHA such as purple sulfur bacteria and purple non-sulfur bacteria. PHB homopolymer was found in purple sulfur bacteria while co-polymers such as 3HB and 3HV were found in purple non-sulfur bacteria (Higuchi-Takeuchi et al., 2016). However, from GPC analysis it was observed that photosynthetic purple bacteria produced PHAs with high molecular weight (3,000–994,000 g mol^−1^) which will be favorable for industrial application. The polydispersity index range was found between 1.5 and 6.7 Mn (Higuchi-Takeuchi et al., 2016). A Horizontal tubular photobioreactor was installed in an Energie-Versorgung Niederosterreich AG power company present in Austria for PHB production. Here, one ton of CO_2_ was used as feed for cyanobacteria and produced 115 kg PHB and biogas up to 320 m^3^ (Salehizadeh et al., 2020). Thus, this strategy will enhance CO_2_ assimilation capacity and could be a promising solution towards large-scale PHB production. Cyanobacteria can efficiently adapt to any environmental condition, which suggests that this group of microorganisms does not require any energy-rich source for their growth, and therefore, they can be exploited for the generation of valuable byproducts such as PHAs and carbohydrates (Parmar et al., 2011; Arias et al., 2020).

**TABLE 1:** Polyhydroxyalkanoates production from CO_2_.

Organism	Pathway	Limitation	Type of bioreactor	Substrate	PHA (mol%)	PHA productivity (g/L/h)	Incubation period	References
PHA production from CO_2_ and H_2_ oxidizing bacteria								
*Cupriavidus necator* DSM 545	PHA synthesis pathway	Nitrogen	Stirred tank reactor	CO_2_	72	0.365	5 days	Garcia-Gonzalez and De wever, (2018)
			Batch	CO_2_	51	0.87	6 days	Ghysels et al. (2018)
*Cupriavidus eutrophus* B-10646			Batch	CO_2_:O_2_:H_2_	85	0.45	3	Volova et al. (2013)
*Bacillus cereus* SS105			Continuous stirred tank reactor	CO_2_	55	NA	NA	Maheshwari et al. (2018)
*Wautersia eutropha* H16 and B5786	Calvin cycle	Nitrogen	Shake-flask	CO_2_ + valeric acid	10	6.1	2	Volova et al. (2008)
*Ralstonia eutropha* B5786			Batch	CO_2_	51	NA	NA	Volova and Voinu, (2003)
				CO_2_ + valeric acid	20	NA	NA	Volova and Kalacheva, (2005)
*R. eutropha*		Oxygen		CO_2_	38	NA	NA	Khosravi-Darani et al. (2013)
*Synechocystis* sp*.* PCC 6803	Genetic engineering approach	Balanced growth	Fed-batch	CO_2_	0.5	0.533	21	Wang et al. (2013)
	Deletion of pirC (regulatory protein of intracellular glycogen and PHB pool) and introduction of phaAB from C. necatorCultivation in CO2 + light with supplementation of acetat	Nitrogen		CO_2_ + acetic acid	81	NA	28	(Koch et al., 2010)
	Insertion of *agp* gene			CO_2_ + acetic acid	18.6	0.00459	NA	Gupta et al. (2013)
	Expression of chromosomal *sigE*			CO_2_	1.4	NA	NA	Blankenship, (2010)
	Expression of PHA synthase from *Microcystis aeruginosa*			CO_2_	7	NA	NA	Wijffels et al. (2013)
	Expression of *xfpk* from *Bacillus breve*			CO_2_	12			Vermaas, (2001)
	Enhancing acetyl-CoA levels	Nitrogen and phosphorous		CO_2_	12	12	0.232	Carpine et al. (2017)
	Trans conjugant cells bearing *pha* genes expression			CO_2_	7	0.98	NA	Hondo et al. (2015)
*Synechococcus* sp*.* PCC7942	Expression of PHA synthase from *Alcaligenes Eutrophus*	Nitrogen		CO_2_ + acetic acid	27–35	NA	NA	Nikel et al. (2006)
	Expression of PHA synthase from *Cupriavidus necator*	Acetate and nitrogen		CO_2_	25	NA	NA	Takahashi et al. (1998)
*Spirulina platensis* UMACC 161	Not available	Nitrogen		CO_2_ + acetic acid	10	NA	10	Toh et al. (2008)
*Nostoc muscorum* TISTR 8871	Not available			CO_2_	0.8	1.73	20	Tarawat et al. (2020)
*Oscillatoria jasorvensis*	Not available			CO_2_ + acetic acid	10	1.1	20	Tarawat et al. (2020)
*Synechococcus* sp*.* PCC 7002	GABA shunt incorporation	None		CO_2_	4.5			Zhang et al. (2016)
*Synechocystis* sp.	Incorporation of acetoacetyl-CoA reductase binding site			CO_2_	35	0.263	5	Wang et al. (2018)
*Synechocystis* sp. PCC 6714	Not available	Nitrogen and phosphorous		CO_2_	16	0.59	14	Kamravamanesh et al. (2017)
*Synechococcus* sp. PCC7002	Expression of *recA* gene from *Escherichia coli*			CO_2_	52	NA	NA	Akiyama et al. (2011)

PHA, Polyhydroxyalkanoate.

HB, Hydroxybutyrate.

HV, Hydroxyvalerate.

### 2.1 Strategy to increase the cyanobacterial PHA production: A genetic engineering approach

*Synechocystis* species are the model organisms studied for various value-added chemicals and biomaterial production (Price et al., 2020). They have been engineered to produce both PHAs and hydrogen. Overexpression of PHB genes in *Synechocystis* sp. PCC6803 increased PHB content. Here, the promoter of the RuBisCO gene was successfully employed for the expression systems in *Synechocystis* sp. PCC6803. Up regulation of RuBisCO activity in the presence of CO_2_ helps to enhance PHB production (Yu et al., 2013). *C. necator* PHA synthase gene was expressed in *Synechococcus* sp. PCC7942 and produced 25% PHB as compared to the non-recombinant strain under phototrophic and heterotrophic conditions (Price et al., 1998). PHA genes were expressed from *C. necator* in *Synechocystis* sp. PCC7002 and produced up to 52% PHA under heterotrophic conditions (Akiyama et al., 2011). *Synechocystis* sp. PCC 6803 was optimized to produce both the (S) and (R) configurations of 3-hydroxybutyrate by inserting two additional pathways that were introduced and produced 533.4 mg/L of PHB (Wang et al., 2013) (Table 1). Recently, *Synechocystis* sp. PCC6803 was engineered to enhance PHA production from CO_2_. Here, the focus was trying to engineer central carbon metabolism, two different target regions, phosphotransacetylase, and acetyl-CoA hydrolase were deleted in *Synechocystis* sp. PCC6803 to see the effect on PHB production. In addition, *Bifidobacterium breve* when expressed with heterologous phosphoketolase produced 12% of PHB content and 7.3 mg/L of productivity (Carpine et al., 2017) (Table 1). Microbial mutualism is beneficial for enhanced PHB production. 2.5-fold of PHB was produced by *Synechocystis* sp. PCC 6714 with a yield of 37% when compared with the wild-type bacteria (Kamravamanesh et al., 2018). Genetically engineered cyanobacteria *Synechocystis* sp*.* generated 1.84 g/L of PHB in 10 days by utilizing CO_2_ with a productivity rate of 263 mg/L/day. Here, acetoacetyl-CoA was found to be the main bottleneck for the enhanced production of PHB (Wang et al., 2018). *Cyanobacterial* expression vectors containing *pha* genes were constructed in *Synechocystis* sp. PCC 6803 and PHB productivity reached 12-fold higher as compared to the wild-type bacteria (Hondo et al., 2015). *Synechococcus* sp. PCC7942 harboring the gene from *Alcaligenes eutrophus* was able to produce 25% of PHB (Nikel et al., 2006). Supplementation of 10% CO_2_ and poultry litter with an aeration rate of 0.1 vvm to *Nostoc muscorum Agardh* produced 774 mg/L of PHAs (Bhatti and Mallick, 2015). *Arthrospira* (*Spirulina*) *platensis* MUR126 co-produced phycocyanin and polyhydroxybutyrate (PHB) under photoautotrophic and mixotrophic condition. Additional supplementation of CO_2_ enhanced the PHA content up to 33% with 23% biomass and 30% phycocyanin.

### 2.2 Physical properties of PHAs derived from CO_2_

The material properties of cyanobacterial PHAs are gaining interest because of their biodegradation properties. Upon degradation, microorganisms break the ester bond and degrade the polymer into oligomers and monomers, whereas petroleum-based synthetic plastics are very difficult to degrade and pollute in the environment. PHB has material properties that are similar to synthetic plastics such as crystallinity, tensile strength, and melting temperature. However, PHB is more brittle than synthetic plastics. The incorporation of 3HV into the PHB chain reduces its brittleness and increases its elasticity, and it becomes tougher and more flexible. This feature makes biopolymers suitable candidates for industrial and biomedical applications. *Aulosira fertilissima* produced PHB and P (3HB-co-3HV), with 155–174°C melting temperature and 0.6–5.5°C of the glass transition temperature. The extracted PHB and P (3HB-co-3HV) also have Young’s modulus ranging from 1.2-3.4 to GPa, while elongation at break property varies from 4.9 to 87.2% (Singh et al., 2017).

## 3 PHA production by hydrogen-oxidizing bacteria

Hydrogen-oxidizing bacteria are the defined group of bacteria that utilize H_2_ and O_2_ as an electron donor and acceptor with the help of ribulose biphosphate for fixing CO_2_ within the bacterial cell (Salehizadeh et al., 2020). *A. eutropha* is capable of converting reduce form of CO_2_ to PHA under different H_2_/CO_2_ stochiometric ratios*.* In addition, *R. eutropha* can produce PHB up to 75% (Khosravi-Darani et al., 2013) (Table 1). The autotrophic culture of *C. necator* was also checked for PHB production. The utilization of CO_2_ led to the production of up to 72% PHB with 60 g/L of dry cell mass (Garcia-Gonzalez and De Wever, 2018). Fed-batch cultivation of the PHA copolymer resulted in 65 g/L cell dry weight and PHBV of up to 27 mol% (Garcia-Gonzalez and De Wever, 2018). The co-utilization of CO_2_ and valeric acid produced 1.14 g/L of P(3HB-co-3HV) by *C. necator* DSM 545 under mixotrophic conditions. Here, CO_2_ was continuously sparged to prevent the accumulation of substrate in the medium (Ghysels et al., 2018). Acetic acid can indeed act as an indirect reservoir of CO_2_ for the production of both PHA homo and co-polymer. The consumption of acetic acid was found to be favorable for *C. necator* growth and PHA production.

## 4 Bioconversion of CH_4_ to PHA

Worldwide, CH_4_ stands as the second most greenhouse gas agent which contributes 20% towards climate change and traps atmospheric heat about 25 times higher than CO_2_ (Mounasamy et al., 2020). Sixty-three percent of CH_4_ is generated from anthropogenic activities and the remainder from coal mining, land filling, anaerobic wastewater treatment (Strong et al., 2016). In natural gas, methane counts for 90%. Due to the current world wide demand it is estimated to increase up to 44.0% by 2040 (Liu et al., 2020). Currently, CH_4_ accounts for 18% of the total greenhouse gas emissions (EU-28) (Cantera et al., 2018a). This situation raises concerns about global warming and has compelled researchers to look for novel approaches that can help to reduce greenhouse gases and environmental pollution. The production of PHB from CH_4_ may act as a sequestration process, and the biotechnological approach could be the best solution for the abatement of methane because it is cost-effective and has a low impact on the environment. Such approaches are mainly based on the biocatalytic action of microorganisms known as methanotrophs that convert CH_4_ into CO_2_ and H_2_O using O_2_ as an electron acceptor (Cantera et al., 2018a; Cantera et al., 2018b). Methanotrophs can grow on C_1_ carbon compounds, and some can utilize multicarbon sources as an energy source in specific environments (Semrau, 2011). The well-studied methanotrophs are aerobic methane-oxidizing bacteria that are categorized as γ-proteobacteria (Type I) and α-proteobacteria (Type II). For carbon assimilation, they follow the ribulose monophosphate pathway (RUMP) and the serine cycle (Pieja et al., 2017). The key enzyme for CH_4_ oxidation is methane monooxygenase which can be found in soluble or particulate forms. Methanogenesis requires two reducing equivalents for the oxidation of CH_4_ into CH_3_OH, where anaerobic methanotrophic archaea and non-methanotrophic bacterial consortia conduct the anaerobic methane oxidation.

### 4.1 PHA biosynthesis by methanotrophic route

Bioconversion of CH_4_ reduces nitrate, sulfur, and metals with the help of methyl-CoA reductase through reverse methanogenesis (Haynes Gonzalez, 2014; Lawton and Rosenzweig, 2016). Efficient PHB production was found in Type II methanotrophs that follow the serine pathway. *Methylosinus trichosporium* OB3b, *Methylocystis paravus, Methylosinus trichosporium* IMV3011, *Methylosinus sporium, Methylocystis hirsute, Methylocystis* spp. GB25 MTS, and *Methylocella tundae* are the most efficient PHB producers (Liu et al., 2020) (Table 2).

**TABLE 2:** Polyhydroxyalkanoate production from CH_4_ and CH_3_OH.

Organism	Pathway	Condition	Reactor type	Substrate	PHA (mol%)	References
PHA production from CH_4_						
*Mithylocystis hirusta*	Serine pathway	Manganese/potassium/nitrogen	Bubble column bioreactor	CH_4_	28	Garcia-Gonzalez and De wever, (2018)
		Nitrogen	Bio filter	CH_4_	45–50	López et al. (2018)
*Methylocystis hirsuta *CSC1			Fed-batch	CH_3_OH	45	Bordel et al. (2019)
*Methylocystis.* sp. GB 25		Potassium/sulfur/ferrous	Stirred tank reactor	CH_4_	33.6	Helm et al. (2008)
					32.6	
					10.4	
		Phosphorous		CH_4_	48	Helm et al. (2006)
		Ammonia/phosphorous/magnesium		CH_4_	51	Wendlandt et al. (2005)
		Nitrogen/phosphrous/magnesium		CH_4_	51	Wendlandt et al. (2001)
					47	
					28	
*Methylocystis* sp. WRRC1		Nitrogen	Serum bottle	CH_4_ + *n-*pentanol/valerate	54	Cal et al. (2016)
*Methylocystis parvus* OBBP		None		CH_4_ + valeric acid	42	Myung et al. (2016)
		Nitrogen		CH_4_ + valeric acid	40	Myung et al. (2015)
*Methylocystis parvus*			Serum bottle	CH_4_/citric acid	30	Zhnag et al. (2008)
*Methylosinus trichosporium*				CH_4_	100	Van der Ha et al. (2012)
*Methylocystis parvus* OBBP			Bioreactor	CH_4_	70	Asenjo and Suk, (1986)
			Serum bottle	CH_4_	49.4	Sundstrom and criddle, (2015)
				4-HB and 5-HV	75	Myung et al. (2017)
			Fluorocarbon emulsion stabilizer	CH_4_	35	Myung et al. (2016)
*Methylosinus trichosporium* OB3b			Batch	CH_4_	29	Rostkowski et al. (2013)
*Methylocystis parvus* OBBP					60	
*Methylosinus trichosporium* OB3b		Nitrogen		CH_4_	38	Pieja et al. (2011)
*Methylocystis parvus* OBBP					36	
*Methylocystis* 4222					25	
*Methylosinus trichosporium*				CH_4_	20–25	Scott et al. (1981)
Methanotrophs				** **CH_3_OH/CH_4_	12–40	Zhang et al. (2008)
Methanotrophic–heterotrophic group			Serum bottle	CH_4_	43–45	Zhang et al. (2018)
		None	Mini Bench top reactors	CH_4_	2.5	Chidambarampadmavathy et al. (2017)
				CH_4_	3	
Methanotrophic– heterotrophic group			Serum bottle	CH_4_	2.5–8.5	Karthikeyan et al. (2015)
Mixed methanotrophic communities		Nitrogen	Stirred tank reactor	CH_4_	20–40	Pfluger et al. (2011)
*Methylotrophic consortium*	Ribulose monophosphate pathway	Nitrogen	Two-phase partition bioreactor	CH_4_	34	Zúñiga et al. (2011)
*Methylobacterium organophilum*					38	
*Methylosinus trichosporium* OB3b			Serum bottles	CH_4_	52	Zhnag et al. (2019)
*Methylosinus*	Ribulose diphosphate pathway		Batch	CH_4_	25	Dalton, (1980)
PHA production from CH3OH						
Methylobacterium extorquens AM1	Ribulose monophosphate pathway	Co^2+^	Serum bottles	CH_3_OH	95.7	Orita et al. (2014)
*Methylocystis* sp. GW2		None		CH_3_OH + valeric acid	40	Yezza et al. (2006)
*Methylobacterium extorquens*		Nitrogen	Bioreactor	CH_3_OH + 5-hexanoic acid	71	Höfer et al. (2010)
*Methylobacterium extorquens*		None	Stirred baffled fermentor	CH_3_OH	33–30	Bourque et al. (1995)
*Methylobacterium extorquens* K		None	Serum bottles	CH_3_OH + n-amyl alcohol	62	Ueda et al. (1992)
*Methylobacterium extorquens* DSMZ1340		Nitrogen	Bioreactor	CH_3_OH	65	Mokhtari-Hosseini et al. (2009a)
*Methylobacterium extorquens* DSMZ1340			Fed-batch	CH_3_OH	46	Mokhtari-Hosseini et al. (2009b)
*Methylobacterium organophilum*	Serine pathway	Phosphorous		CH_3_OH	52	Kim et al. (1996)
*Mycoplana rubra*	Not reported		Batch	CH_3_OH	80	Sonnleitner et al. (1979)
*Methylobacterium* sp. V49	Not reported	None		CH3OH	11	Ghatnekar et al. (2002)
				Sucrose	28	
				Glucose	53	
				Lactose	40	
				Fructose	25	
*Methylobacterium rhodesianum*		None	Bubble column bioreactor	CH_3_OH	50–45	Babel, (1992)

PHA, Polyhydroxyalkanoate.

HB, Hydroxyalkanoate.

HV, Hydroxyvalerate.

In a sequential step, methanotrophic bacteria oxidize CH_4_ to CO_2_ with intermediates such as methanol, formaldehyde, and formate. CH_4_ is oxidized to methanol by particulate and soluble methane monooxygenase. Formaldehyde is produced in the second step of oxidation. However, a high concentration of reducing equivalents is, therefore, required to convert cytosolic formaldehyde to formate during the 2nd step of oxidation. NADH^+^ specific and cytochrome-linked enzymes are involved in this process. In the case of type-I methanotrophs, formaldehyde fixation is catalyzed by 3-hexulosephosphate synthase and formed (D-arabino)-3-hexulose-6-phosphate, which in turn converted to different intermediates and CO_2_. Here, for enzyme activation Mg^2+^ and Mn^2+^ co-factors are used. In contrast, type-II methanotrophs activate formaldehyde oxidation by pterin cofactor, which is catalyzed by tetrahydrofolate (H_4_F) enzymes. Tetrahydrofolate enzymes supply the formaldehyde acceptor known as N^5^, N^10^ methylene-H_4_F, which supports the chemical reaction between formaldehyde and glycine to form serine. Subsequently, depending on nutrient availability and CO_2_ production type-II methanotrophs move towards TCA cycle or PHB production pathway (Figure 1).

The equation for the PHB cycle is

$$2{CH}_{4}+3O_{2}+6{NADH}_{2}+2{CO}_{2}\;\to \;C_{4}H_{6}O_{2}\;\left(PHB\;monomer\right)+{FPH}_{2}$$ (1)

$$8{CH}_{4}+12O_{2}+FP\;\to \;C_{4}H_{6}O_{2}\;\left(PHB\;monomer\right)+4{CO}_{2}+12ATP+{FPH}_{2}$$ (2)

where FP and FPH_2_ is oxidized and reduced flavoprotein, respectively.

### 4.2 Strategy for enhanced PHA production from CH_4_

Several methanotrophs utilize CH_4_ and produce PHB under nutrient-limiting conditions (Laycock et al., 2013). Co-substrate addition might be helpful for higher PHA copolymer production. Furthermore, there could be a combination of biological and chemical approaches that can help to produce PHAs with improved properties. The quality of methanotrophic PHA remains compromised in large-scale industrial production and its use over synthetic plastics. The addition of C_1_ substrates such as formate to CH_4,_ was capable of slowing the PHB consumption of *Methylocystis parvus* OBBP (Pieja et al., 2011). Under some conditions, CH_3_OH maintains methylotrophic activity (Khosravi-Darani et al., 2013). *Methylotrophic trichosporium* produces PHB by utilizing CH_4_ and CH_3_OH under short non-axenic conditions. Here, the addition of 0.1% (v/v) CH_3_OH maintained the oxidation level of CH_4_ (Zhang et al., 2008). The experiments were operated in a two-stage cultivation step to maintain the growth and produce up to 0.6 g/L of PHB. Other studies have proposed that the addition of methanol and other C_1_ substrates to CH_4_ enhances methane bioconversion, cell biomass, and PHA content (by 10–35% (w/w)) (Zhang et al., 2008) (Table 2). The addition of citric acid also resulted in enhanced PHA production because it acts as an inhibitor for the TCA cycle and accumulates twice amount of PHA as the control, even under sufficient nutrient conditions. PHB production increased from 12 to 40% (w/w) with M_w_ up to 1.5 × 10^6^ Da (Zhang et al., 2008).

Furthermore, the supplementation of volatile fatty acids such as acetic acid, propionic acid, butyric acid, and valeric acid to CH_4_ enhanced PHB production in *Methylocystis hirsuta* with a yield of 10–30% (López et al., 2019) (Table 2). The co-utilization of valerate and methane led to 3HV production up to 20–40 mol% by *Methylocystis hirsuta*, *Methylocystis parvus* OBBP, and *Methylosinus trichosporium* (Cal et al., 2016; Myung et al., 2016). However, the co-utilization of propionate and n-pentanol did not significantly affect the 3HV content in *Methylocyctis* species (Cal et al., 2016; Myung et al., 2016) (Table 2). Interestingly, when co-fed under copper limitation, Valerate and CH_4_ enhanced the PHA and its co-polymer content up to 78% and 50 mol% (Cal et al., 2016). The addition of ω-hydroxy alkanoates to CH_4_ resulted in new tailor-made co-polymers by *Methylocystis parvus*, which has a molecular weight ranging from 4.5 × 10^5^ to 1.5 × 10^6^ Da (Myung et al., 2017). Here, the composition of PHA co-polymers consists of P (3HB-co-4HB), P (3HB-co-5-HV-co-3HV), and P (3HB-co-5HV-co-3HV) (Myung et al., 2016) (Table 2).

Most methanotrophic PHA production has occurred using obligate type II methanotrophs *via* batch fermentation. Its main function is to use pure CH_4._ Thus, many studies have attempted to design a bioreactor for simultaneous CH_4_ mitigation and PHB production. *Methylocysitis* was cultivated in a sequencing batch reactor and a bubble column bed reactor for the accumulation of PHB up to 20–25% (w/w) and 40–50% (w/w), respectively (Pieja et al., 2011; Garcia-Gonzalez and De wever, 2018) (Table 2). PHB production was higher in the case of the bubble column bed reactor. This might have occurred because of the internal gas-recycling system, which allows CH_4_ mass transfer compared to the sequencing batch reactor. PHB production by *Methylocystis* sp. GB25 occurred in a two-step phase in the STR with PHB production in batch fermentation (López et al., 2018) (Table 2). Microalgae *Scenedesmus* sp. and *Methylocystis parvus* OBBP converted CH_4_ and CO_2_ from synthetic biogas into PHB in a two-stage process (Van der Ha et al., 2012) and PHB accumulation was also studied in *Methylocystis hirsuta* by exploiting CH_4_ from biogas as a source (López-Neila, 2017). Another study revealed that *Methylocycstis*-enriched cultures utilize trace amounts of ethane, propane, and butane in natural gas and produced 42% (w/w) of PHB (Myung et al., 2016) (Table 2). Thus, utilization of co-substrates and up-gradation of reactors is desirable to raise PHA production/yield.

## 5 PHA production by CH_3_OH

Methanol can be generated not only from industrial waste gas but also from biomass and CO_2_ hydrogenation process (Borisut and Nuchitprasittichai, 2019). CH_3_OH can be used as feedstock in both biological and chemical industries by methylotrophs for the sustainable production of value-added chemicals (Liao et al., 2016). Under environmental conditions, CH_3_OH is present in liquid form, thus, easy to handle and bio convert to PHA production. The application of methylotrophic bacteria facilitates PHA production from CH_3_OH.

Among others, *Methylobacterium* spp. is the most widely studied organism for PHB synthesis through the serine pathway. NH_4_^+^, Mg^2+^, PO_4_^3-^, and K^+^ are the major deficiencies studied during the accumulation of PHB (Khosravi-Darani et al., 2013). *Methylobacterium extorquens* were studied for PHB and its co-polymer production from CH_3_OH (Bourque et al., 1992). The optimization of fed-batch fermentation led to a cell dry weight up to 115 g/L and a polymer content of up to 46% (w/w) (Bourque et al., 1992) (Table 2). The maximum PHB production using CH_3_OH as carbon feedstock was found to be 0.18 g/g. A recent study suggested that the addition of a co-substrate such as valeric acid, n-amyl, or propionic acid to CH_3_OH could be helpful in the higher production of PHB and its co-polymer (Strong et al., 2016). Enhanced PHB production of up to 136 g/L was achieved by using computer-controlled fed-batch fermentation of CH_3_OH with 66% cell dry weight using was obtained. It has been proposed that genetic engineering may enhance PHB productivity and yield. *Methylobacterium extorquens* DSMZ 1340 utilized methanol for the production of PHB. The deficiency of MgSO_4_ and (NH_4_)_2_SO_4_ in the medium stimulates PHB production, which increased up to 62.3% of total dry cell mass by *Methylobacterium extorquens* DSMZ 1340. After 35 h of fed-batch fermentation, the yield of PHB was 2.8 g/L/h and 0.98 g/L/h (Mokhtari-Hosseini et al., 2009a). In contrast, the growth of *Methylobacterium trichosporium* IMV3011 on CH_3_OH was tested at a feeding rate of 0.1% five times during fermentation. Hence, the concentration of PHB increased to 47.6% with 2.91 g/L cell dry weight. The addition of malic acid at the rate of 0.2 g/L enhanced to 58.5% of PHB production, with 3.32 g/L dry cell mass. Various reports have suggested that PHB production can be enhanced after the addition of acetyl-CoA, malic acid, and citric acid (Xin et al., 2011; Khosravi-Darani et al., 2013). Similarly, the addition of 0.12 g/L of NH_4_^+^ produced 7.04 g/L of PHB by *Methyloligella halotolerans* from methanol with a molecular weight of 8,000–10,000 kDa (Ezhov et al., 2017)*.* Under the nutrient limitations of nitrogen and magnesium*, Methylobacterium extroquens* DSMZ 1340 produced 9.5 g/L of PHB with a cell dry weight of 65.3% and a yield of 0.16 g/g by utilizing CH_3_OH (Mokhtari-Hosseini et al., 2009b) (Table 2).

Moreover, *Methylobacterium extorquens* DSMZ produced 44% (w/w) of PHB by fed-batch cultivation. Recombinant technology helped to produce PHA copolymers such as 3HB, 3HV, and 3HHx within the polymer in *Methylobacterium extorquens* AM1 (Orita et al., 2014) (Table 2). The present study reported that C_O_^2+^ concentration in the medium influences cell growth and PHA productivity*.* A low concentration of C_O_^2+^ helped to achieve 3HV content up to 6 mol%. Furthermore, the *pccA* gene deletion increased the 3HV fraction in terpolymers (Orita et al., 2014) (Table 2).

*Methylobacterium extorquens* AM1 utilized CH_3_OH as a sole source of carbon and produced PHB up to 149 g/L (Suzuki et al., 1986). *Methylobacterium extorquens* ATCC55366 synthesized up to 46% (w/w) PHB by utilizing CH_3_OH. Moreover, under oxygen-limiting conditions and addition of 0.01 g/L of CH_3_OH led to the production of PHB with high molecular weight (900–1,800 kDa). The composition of media was found to be a significant approach for higher cell biomass and PHB production. The cheaper cost of methanol could, therefore, reduce the cost of PHA production when compared to pure sugar, but the yield is low for other substrates (Höfer et al., 2010) (Table 2). Moreover, the tolerance of organisms should be improved for industrial applications (Zhag et al., 2018).

## 6 PHA production from CO

Several thermal power plant and steel plant industries generate large quantities of carbon monoxide. For example, a South Korean steel company (POSCO) generates 60 million Nm^3^ of CO/day (Hwang et al., 2020). CO can be utilized to produce value added products. However, the processes have several limitations. Being an inert gas, the efficiency of this process is low, and the risk of the explosion has limited the use of CO (Choi et al., 2020a). It is toxic to several microorganisms but some microbes can utilize CO.

*Acetobacterium woodii* produced PHB using CO as a substrate in a two-phase whole-cell bio-catalytic operating system. *A. woodii* utilized CO and produced 53.1–60.7 mM of formate in the first step. In the second step, engineered *Methylobacterium extorquens* subsequently utilized formate, and the PHB content improved by up to 2.24%. The two-stage concept for the bioconversion of CO to PHB offers an effective solution for CO utilization (Hwang et al., 2020). Although, the interest in bioconversion of CO to PHA has increased recently, however, it is still in a nascent stage. Thus, much insight is required about the underlying mechanism. Once deciphered, it would be an asset for the biotechnological industries (Figure 3).

## 7 PHA production from formate

Bioconversion of C1 feed substrates to value-added compounds have attracted researcher owing to the environmental pollution and the shale gas revolution (Durre and Eikmanns, 2015; Humphreys and Minton, 2018; Sun et al., 2019). Electrochemical reduction of carbon dioxide or syngas hydration lead to the generation of formate (HCOO^−^) which is regarded as environmentally friendly and sustainable microbial feed substrate (Agarwal et al., 2011; Schuchmann and Muller, 2013; Hwang et al., 2015; Shinagawa et al., 2018; Hwang et al., 2020). CO_2_ is reduced to formate, methane, carbon monoxide, and ethylene (Whipple et al., 2010). Formate can be used as fuel in fuel cells (Joo, 2008; Enthaler et al., 2010). CO_2_ reduction by the electrochemical reaction can produce formic acid with high faradaic efficiency. Also, one oxygen-stable whole-cell biocatalyst is used to produce formate (Kortlever et al., 2015). It can act as a good electron mediator that has stability, non-toxicity, good permeability. Methylotrophs, non-pigmented *Pseudomonas*, facultative autotrophs, heterotrophs, and hypomicrobia are capable of growing on formate through serine pathway or ribulose monophosphate (RuMP) pathway. (Hwang et al., 2015) (Figure 3).

In bacteria, formate is oxidized to carbon dioxide by generating NADH (Berrios-Rivera et al., 2002; Yishai et al., 2016; Barin et al., 2018). Several reports are found on the utilization of formate by *Escherichia coli* using formate assimilating pathways. However, due to slow bacterial growth, the industrial application has been limited (Kim et al., 2019; Bang et al., 2020; Kim et al., 2020). On the other hand, *Methylorubrum extorquens* can able to utilize formate through the tetrahydrofolate pathway and serine cycle (Yishai et al., 2016; Hwang et al., 2020). Metabolic engineering of *Methylorubrum extorquens* has enhanced the product yield of various bioproducts from C1 feed such as PHAs, proteins, dicarboxylic acids (Bélanger et al., 2004; Choi et al., 2008; Hofer et al., 2010; Orita et al., 2014; Sonntag et al., 2014, 2015). One study reported that *Methylobacterium extorquens* utilized formate and produced P(3HB-co-3HV) with 8.9% HV content. Here, *bktB, phaJ1,* and *phaC2* were heterologously expressed and effectively converted propionyl-CoA into 3-HVCoA (Yoon et al., 2021). *Methylobacterium extorquens utilizes* formate through the Foc A transporter (Lü et al., 2011; Hwang et al., 2020).

## 8 Technical glitches with their promising solutions in the path of viable PHA production from C1 sources

The effect of PHA-producing organisms on a commercial scale is indicated by the PHA yield, organism growth rate, and rate of PHB accumulation. The productivity of PHA is directly proportional to the price of the polymer. The conversion of CO_2_ to PHA depends mainly on the bacterial cell growth rates and cell densities of the organisms. The unavailability of a mass cultivation system is hindering the growth of commercial production of PHA from CO_2._ Cyanobacterial mediated PHA production is generally influenced by abiotic factors, and operational parameters (Singh et al., 2017; Singh et al., 2019). The cyanobacterial production of PHA varies significantly, and there are high chances of the simultaneous production of other value added byproducts for example, proteins and pigments. The process parameters should be constant to achieve a high PHA yield. The commercial cultivation of PHA can be achieved in two ways: 1) Photobioreactor (closed system) and 2) pond culture systems (open) (Koller and Marsalek, 2015). Some challenges remain, for example, 1) improved photoautotrophic PHA production, 2) enhanced bacterial cell biomass in the photobioreactor, 3) photobioreactors modification for scale-up purpose, 4) Optimized downstream processing, and 5) CO_2_ uptake capacity (Drosg et al., 2015). An ideal photobioreactor system can maintain a phototrophic culture system for the production of valuable bio-products. An advanced photon system was operated for cultivation system with an average temperature range of 15–60°C (Singh et al., 2017; Singh and Mallick, 2017). Maintaining a monoculture and its productivity is the main problem of the system. Also, compared to open pond systems photobioreactors are more expensive. There are several uncertainties due to the underdevelopment of photobioreactors and open pond systems (Singh et al., 2017). The complexity found in the harvesting and processing of biomass limits the growth of PHA towards commercialization. Harvesting is an approach that creates a bottleneck for commercial scale. Different techniques have been employed for harvesting cyanobacterial biomass, for example, filtration, centrifugation, flocculation, and gravity settling. However, because of the small size, low concentration, and colloidal stability of cyanobacterial biomass, the harvesting process is difficult to recover (Xia et al., 2017). Furthermore, the harvesting cost is 20–30% of the entire process. After the harvesting process, it is necessary to eliminate water from the wet biomass, which helps to further store cyanobacterial feedstock, and therefore, intensive energy is required for the cyanobacteria drying process. A study has reported that drying alone costs 20% of the total production cost. Air-drying is also possible for cyanobacterial biomass production, but it requires a longer time and additional storage place. However, the air-drying process could be replaced by solar and wind energy (Parmar et al., 2011).

The biotechnological approach based on CH_4_ bioconversion is promising and imminent, but there are some physical and biological limitations. Being a hydrophobic gas pollutant, CH_4_ has poor solubility in water, which results in a low elimination rate and poor biomass concentration. Uptake of CH_4_ gas to bacterial communities creates problems, and there is a need to search for innovative strategies that can make the process easier and more successful (Kraakman et al., 2011). Mechanical stress of the bioreactor limits the growth of methanotrophs by hindering the agitation rate (Cantera et al., 2018b). For this reason, conventional bioreactors remain limited (Muñoz et al., 2007; Kraakman et al., 2011; Estrada et al., 2012). Furthermore, an increase in both investment and operating costs is influenced by a low gas-liquid concentration gradient (Estrada et al., 2012). For microbial conversion of waste gas streams (rich in CH_4_) into valuable bio-products, suspended growth bioreactors are found suitable (Muñoz et al., 2007). Suspended growth membrane diffusion and pressurized bioreactors have been developed to enhance mass transport and require low-moderate energy for operation. Also, through internal gas recirculation, a high mass transfer rate of CH_4_ can be achieved (Estrada et al., 2014). Conversely, some environmental variables, such as culture conditions, pH, temperature, dissolved oxygen (O_2_) concentration, CH_4_ and O_2_ ratio, cultivation time, and the composition of cultivation media also play a major role in the CH_4_ conversion process (Semaru et al., 2010; Liao et al., 2016). The addition of a high salt concentration with stable pH values avoids contamination of the whole process. Co-substrates addition to the CH_4_ conversion process could be applied to tailor the quality of the byproducts. One study demonstrated dioxygenase enzyme for methane activation by oxidizing two methane molecules simultaneously. This approach could enhance process performance (Liao et al., 2016). In this context, “omics” technology will soon contribute to improving methanotroph-based biorefinery. Several companies, such as the Intrexon Corporation and Calysta are conducting more research and investment to find a single methanotroph capable of producing multiple value-added bioproducts (Cantera et al., 2018a; Cantera et al., 2018b). An advanced reactor set-up would certainly be helpful for enhanced bioavailability and conversion of gaseous C_1_ substrates.

The generation of high value-added bioproducts could be achieved by fed-batch cultivation through a controlled nutrient feed strategy in mechanically stirred fermenters to gain high bacterial cell densities (Harding et al., 2007). Syngas contain CO, that has a high affinity towards metal-containing enzymes which makes the fermentation process toxic. To avoid such toxic substances, enzymatic or chemical extraction methods are used. Although the conversion of C_1_ substrates to PHA seems lucrative, several limitations need to be overcome to enhance the economic status of PHA (Singh et al., 2017). An eco-friendly, cost-effective PHA recovery is required from biomass.

### 8.1 Process engineering strategies for PHA production from C1 gases

Using several C1 sources, PHA can be produce in both methylotrophs and autotrophs. However, the overall production cost is comparable to PHA produced from other sugar substrates due to their low utilization efficiency. This strategy can be applied to achieve three objectives: 1) Increasing the PHA content, 2) production of co-polymers, 3) enhancing the mass-transfer rate that affects the final productivity, titer of the PHA production. Several studies have researched the process engineering strategy that aiming to produce various PHAs from C1 substrates. Nutrient limiting conditions favor the production of PHA in microorganisms such as methylotrophic bacteria, *a*-proteobacteria or *Cupriavidus* species (Pérez et al., 2019). However, PHA can be degraded within the cells as a reservoir of carbon or redox balance under normal conditions. *Methylosinus, Methylocystis, Methylobacterium* produce 40% PHA in nutrient limiting conditions while *Cupriavidus* species produce more than 60% (Joo, 2008). Furthermore, along with nutrient strategy feast and famine feeding process could help to achieve high PHA content. The monomeric compositions of PHA make them stiffer and brittle that limits their application in several fields. Incorporation of co-polymers improves the flexibility, lower melting temperature, glass transition temperature, crystalinity of PHA. Thus, several precursors molecules have been employed for the production of PHA co-polymers with desired material properties and physic-chemical properties in methylotrophic and autotrophic strains. Gas-to- liquid mass transfer plays a major role in microbial PHA production from C1 gases. To improve the low mass transfer rate bubble column and vertical tubular loop reactors were employed for the production of PHA by *Methylocystis hirsute* using CH_4_ as a carbon source with a yield of 42.5% and 51.6%. The high cell density of *M. hirsuta* led to the production of 73.4% PHA in the bubble column reactor (Pieja et al., 2011; López et al., 2019; Mozumder et al., 2015).

### 8.2 Metabolic engineering strategies for PHA production from C1 gases

Process engineering approaches have led to the production of high yield PHA from C1 resources. But, the host strain development is still facing challenging. Metabolic engineering of microbial strains for the production of high yield PHA depend upon three objectives: 1) engineering of heterotrophs that cannot utilize C1 sources but can produce PHA, 2) engineering of methylotrophs and autotrophs that do not able to produce PHA, and 3) engineering of the metabolic pathways in methylotrophs and autotrophs that produce PHAs and their co-polymers naturally. When CODH from *Oligotropha carboxidovorans* was introduced in *C. necator* H16, it produced 49.7% PHA when grew in CO as whole carbon source.

There are several microorganisms can grow efficiently on CO_2_ as sole carbon source but cannot produce PHA naturally. Due to the absence of *Pha* operon, Acetogens cannot produce PHA from C1 sources under autotrophic conditions. Although the uptake of CO and CO_2_ led to the conversion of Acetyl Co-A which is the building block of PHA production (Lembruger. et al., 2019; Flüchter et al., 2019). Insertion of codon optimized Pha operon from *C. necator* into *Clostridium autoethanogenum* led to the production of PHA from syngas mixture containing CO and CO_2_ (Joo, 2008).

The attempts to engineer methylotrophic bacteria for the production of value added bacteria have been increasing slowly. Recombinant *M. extorquens* AM1 produced P(99 mol%3HB-co-0.7 mol% 3HV) co-polymer and P(99 mol% 3HB-co-0.3 mol%3HHx) terpolymer under CO^2+^ limited conditions (Lee et al., 2015; Kalyuzhnaya et al., 2015; Pfeifenschneider et al., 2017).

### 8.3 Biotechnological advances of PHA production from C1 sources

Several microbes such as wild type, mesophilic, extremophilic, and genetically modified organisms have been employed for the production of PHA (Koller and Marsalek, 2015). Next-generation industrial biotechnology (NGIB) approaches help to use genetically tailored strains to enhance the industrial production of PHA. To improve the PHA production efficiency, genetic manipulation was tested from substrate uptake and utilization (Povolo et al., 2010; Chen and Jiang, 2018). CRISPR/cas9 genome has been used to enhance the PHA copolymer production. This approach will help to predetermine the monomeric composition of PHA by knocking out some targeted pathways. Also, synthetic biology approaches help to construct artificial metabolic pathways which lead to the production of high molecular weight of PHA and its co-polymers (Zhou et al., 2012). Also for downstream processing, genome editing strategy was found helpful for process involvement (Gamero et al., 2018). Engineered microbes help to convert renewable substrates to PHA is of great importance towards sustainable production (Koller et al., 2008). PHA production from CO_2_ using cyanobacteria as photoautotrophic cell factories has gained attention. CO_2_ from industrial effluent has been used to produce value-added chemicals by microbes at the same time mitigating greenhouse emissions. Optimization of photobioreactor is required to enhance the cyanobacteria cultivation. For optimization, geometric characteristics, mixing behavior, gassing/degassing performance, illumination regime are needed. A recent report revealed that *Synechocytis* sp. inoculated with tubular photobioreactors was found to enhance PHA production (Troschl et al., 2017a). The photobioreactor was up to 200 L. 6 gas-discharge lamps were used with light-dark cycle of 16 h/8 h. With the help of help PAR sensor, photosynthetically active radiation was measured. A degasser was used in the reactor for removal of oxygen. Probes such as CPS11D and COS51D were used to measure oxygen and pH of the reactor. Pure CO_2_ was injected to control pH (Troschl et al., 2017b).

*Methylocystis* sp. utilizes CH_4 to_ produce 0.5 g of PHA that is higher than the heterotrophic PHA production from other carbon sources such as sugars. Gasification of organic waste led to the production of syngas which can be utilized to produce PHA by *Rhodospirillum rubrum* (Revelles et al., 2016).

The NGIB approach uses artificial intelligence that allows low-cost renewable substrates and wastewater for the production of PHA (Chen and Jiang, 2018). PHA production is mainly influenced by the cost of the substrates. Low-cost substrates such as household wastes, activated sludge, shale gas, or syngas could be used to lower the production cost of PHA (Nikodinovic-Runic et al., 2013). Moreover, bioconversion of renewable high substrates to PHA efficiency is the most significant approach to reduce substrate consumption. Seawater could be used as the widely available and inexhaustible water source could be used as the sustainable source to replace limited water source in next-Generation Industrial biotechnology. Extremophiles are more robust as they can sustain under a long continuous operation controlled by artificial intelligence. Artificial intelligence can be achieved by collecting big data from bioprocessing parameters followed by the machine. Extremophiles and their genetically modified strains such as acidophiles, psychrophiles, thermophiles, methanotrophs are found suitable microorganism for NGIB as they grow rapidly, resistant to contamination, and feasibility for genetic engineering (Chen and Jiang, 2018; Yin et al., 2018; Quillaguaman et al., 2006; Sedlacek et al., 2019).

## 9 Diverse applications of PHA

**FIGURE 4:**
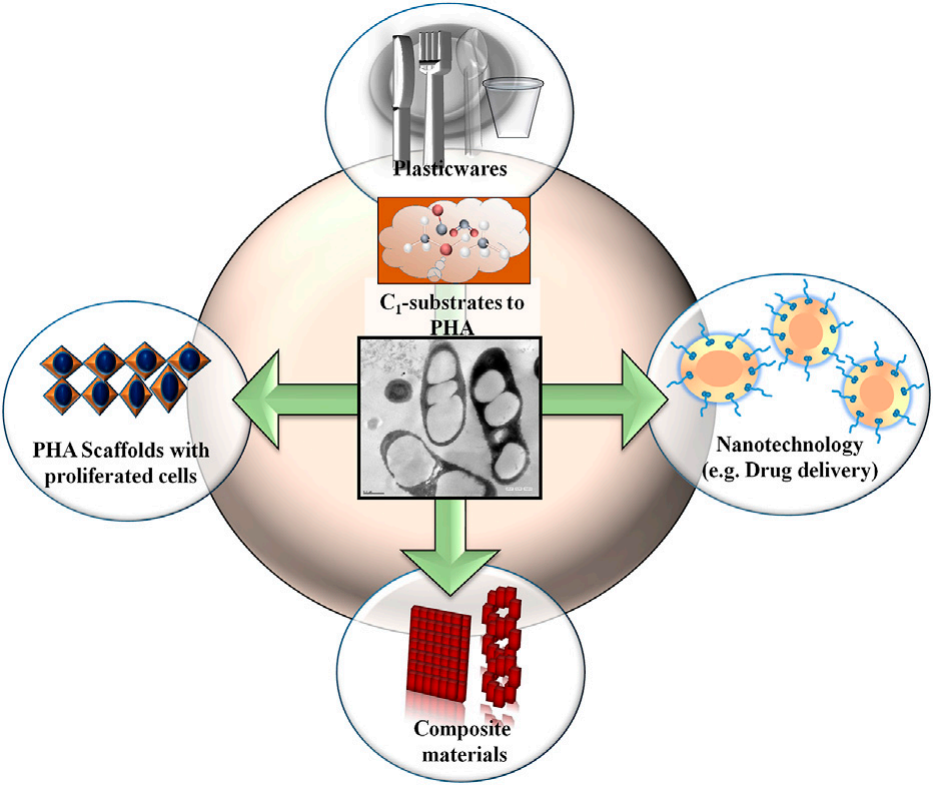
Production of biopolymers from c, substrates. Applications of polyhydroxyalkanoate (PHA) in different field.

PHA polymers may possess more than 160 types of monomers that endow a wide range of physical properties (Choi et al., 2020a; Alcântara et al., 2020). Also, the biocompatibility and biodegradability qualities of PHAs have attracted more attention towards the medical field and therapeutic material industries (Figure 4). Therefore, applications of PHAs range from preparing commodity products to agricultural sectors. PHAs are more suitable agents for biomedical applications because the *in vivo* degradation of PHA is less acidic as compared to polylactic acid and poly (lactic-co-glycolic acid), which cause local necrosis and inflammation (Choi et al., 2020b). The biomedical use of PHAs started when surgical sutures were tested using P (3HB). Sutures made of PHAs were implanted in rats and were found to remain active for a long time without any adverse effects (Shishatskaya et al., 2004). Several co-polymers of PHAs were later exploited in biomedical field in the form of heart valve scaffolds, drug delivery agents, surgical sutures, bone marrow scaffolds, stents, cardiovascular patches, orthopedic pins, adhesion barriers, tendon repair devices, wound dressings, and (Gumel et al., 2013; Ray and Kalia, 2017c; Chen and Zhang, 2018). Owing to their biocompatible nature, PHA-based scaffolds such as P(3HB-co-3HV) used in the preparation of laser-perforated biodegradable scaffold films that repair damaged tissue and enable a higher rate of cell adhesion, growth, and migration (Ellis et al., 2011) (Figure 4). The fabrication of carbon nanotubes with PHBHHx nanocomposite film was reported to support osteogenesis process (Wang et al., 2011; Wu et al., 2013) and similarly, pre-osteoblast cells development was also found by the blending of P (3HB-co-3HHx) and polycaprolactone (Puppi et al., 2017). Other unique approaches have recently been developed, for example, PHB with poly-lactide-caprolactone was used for the preparation of electrospun nanofibrous scaffolds to accelerate progression in cell cycle and prevent necrosis (Daranarong et al., 2014), and PHA microspheres were used for proliferating stem cells and osteoblast regeneration (Wei et al., 2018). Conversely, the application of PHA is focused on the regeneration of new bone by employing bone tissue engineering. A blend of 8% 3HV with 30% hydroxyapatite composition had shown suitable mechanical compressive strength with lower inflammatory response and mineralization (Galego et al., 2000). Terpolyester of PHA scaffolds enhanced osteoblast attachment, propagation, proliferation, and differentiation in nerve cells (Li et al., 2005; Wang et al., 2010). Among various PHA polyesters, P(3HB-co-3HV) was shown to be the best for bone tissue regeneration (Yang et al., 2004). The degradative PHA (3-hydroxybutyrate) has been shown to promote the growth of murine osteoblast MC3T3-E1 cells *in vitro*. In addition, *in vivo* studies on ovariectomized rats suggested the role of 3HB in preventing osteoporosis, as revealed by a bone material density and bone biomechanics study (Zhao et al., 2007). A unique PHBHHx-based microgroove surface pattern (10 µm size) influences the interfacial behaviors of bone marrow mesenchymal stem cells and differentially expressed miRNAs in comparison to smooth-surfaced PHBHHx substrates (Wang et al., 2013). PHA also strengthens the skeleton, increases the rate of calcium deposition, and lowers the level of serum osteocalcin (Zhao et al., 2007; Guyot et al., 2020). PHB/chitosan nanofibers have been helpful in cartilage tissue engineering (Sadeghi et al., 2016). These studies demonstrate the beneficial biomaterial properties of PHAs in different medical applications.

A study reported that PHB film was used for cancer detection (Sabarinathan et al., 2018). Thus, the use of PHB films for cancer detection was time-saving and painless compared to biopsy (Sabarinathan et al., 2018). PHA can also be used as a drug carrier. PHA copolymers were used to release anticancer drug 5-fluorouracil (Choi et al., 2020a). Several periodontal disease bacteria were killed by P(3HB-co-3HV) microspheres and microcapsule containing tetracycline [151]. Electrospun fibrous meshes of PHB-polyethylene oxide were used as drug delivery agents for chlorhexidine against some pathogenic bacteria (Meng et al., 2013). Similarly, a blend of PHB-chitosan was used to deliver ketoprofen, which is an antipyretic (Lins et al., 2014). Also, 3HB derivatives of PHA polymers can be used as effective drugs against mitochondrial damage (Zhang et al., 2013). Methyl ester of the 3HB monomer has shown to be effective against Alzheimer’s and Parkinson’s diseases (Camberos-Luna et al., 2016; Camandola mattson, 2017). Hydroxy acid produced from PHA degradation has also been shown to play a major role in calcium ion stimulation in memory enhancers (Raza et al., 2020). 3HB also a degradative product of PHA has an anti-osteoporosis effect (Cao et al., 2014).

Applications of PHA have been further extended through various chemical functionalization approaches. In addition to medical use, PHAs are also suitable for food packaging, cosmetics, and agricultural applications. Produced from municipal biowastes, P (3HB-co-3HV) were electrospun to prepare bio papers for food packaging (Melendez-Rodriguez et al., 2020). Due to the high radical scavenging activity, high water vapor transmission, antioxidant and antibacterial property, PHB film can act as a biodegradable active packaging material. For example, PHB film is used in the wrapping of chilled salmon (Ma et al., 2018). PHA can be used to make straws and bottles. A degradable straw was prepared using Nodax PHA (Danimer Scientific, U.S.A.) in 2018 (Choi et al., 2020a). Recently, Nestle Co. with Danimer Scientific developed biopolymer-based bottles (Choi et al., 2020b). Moreover, several PHAs blended with different biobased materials are used in the cosmetic industry for the preparation of biobased beauty masks and sanitary napkins (Choi et al., 2020b). PHA microplastics can be used in face cleansers and toothpaste as an alternative to synthetic microplastics, which are harmful to live beings, including aquatic animals (Bouwmeester and Hollmanpeters, 2015). Recently, a sun protection product containing PHA micropowder was introduced (Unilever, U.K.) as containing the first biodegradable cosmetic ingredients derived from renewable biomass (Choi et al., 2020a). Several farmers use plastic materials for greenhouse films and protection nets, which are essential for increasing the quality of crops and protecting them from hazards. High-density polythene is a commonly used synthetic plastic material but owing to its non-biodegradability, PHA films are currently being considered. Large quantities of bags are used in the agricultural sector for fertilizers and seedlings, among others (Adane and Muleta, 2011). In this context, PHA bags have many advantages PHA polymer bags do not release any toxic chemicals into the soil.

PHAs can also be helpful in denitrification. Recently, a modified sequencing batch biofilm reactor was designed to treat landfill leachate. Here, ammonia-oxidizing bacteria and denitrifying bacteria are capable of transforming organic carbon compounds into intracellular PHA (Yin et al., 2018). In this reactor, the process of nitrification (removes NH_4_^+^ –N to produce NO_2_^−^ –N), simultaneous nitrification and denitrification (removes NO_2_^−^ –N), and endogenous denitrification (removes residual NO_2_^−^ –N) utilized PHA as a carbon source (Yin et al., 2018). PHA films can be used to prepare biodegradable waste bags, which can contribute to reducing landfills and making the composting process more efficient. PHA films can be used in agriculture and reduce disposal costs (Winnacker, 2019). Recent developments in these areas are promising, and will certainly increase the value of PHA polymers for their useful applications in daily life. PHAs are used in 3D printing implants for humans (Rydz et al., 2019). Chemically modified PHAs such as Polyhydroxyalkanoate-g-poly (N-isopropylacrylamide) has shown thermal responsive hydrophilicties and biocompatibilities at different temperature (Ma et al., 2016). Modified PHA with EU3^+^ and Tb3^+^ possesses intense photoluminescence properties with enhanced hydrophilicity and biocompatibility (Yu et al., 2019a, Yu et al., 2019b). PHAs can be also used as nano-vaccines in drug delivery (Parlane et al., 2012).

Various companies are known for the production of PHAs. TianAN is the largest PHAs production company. PhaBuilder, Medpha, COFCO, and Bluepha have been set up to explore Next-Generation Industrial Biotechnology for low-cost PHAs production. Metabolix company was sold to Korean company CJ. RWDC and Danimer have employed recombinant *R. eutropha*, which can utilize fatty acids for P (3HB-co-3HHx) production. New light and Full cycle companies utilize greenhouse gas, organic wastes respectively for the production of PHAs. Recently, a global organization is known as GO! PHA has been established to produce PHAs (Table 3.).

**TABLE 3:** Industrial companies for PHA production.

Company	Microorganisms	Type of polymer	Production (ton/yr)	References
Tiana Biol. China	*Ralstonia eutropha*	P (3HB-co-3HV)	2000	www.tiananenmat.com Enmat
Danimer Scientific, United States		P (3HB-co-3HHx)	10,000	danimerscientific.com
Cheil Jidang South Korea		P (3HB-co-3HV)	50,000	Biopol
Kaneka, Japan		P (3HBco-3HHx)	5000	www.kaneka.be
Pha Builder, China	*Halomonas* sp	PHA and its co-polymers	1000–10000	www.phabuilder.com
Green Bio, Tianjin, China	*Escherichia coli*	P (3HB-co-4HB)	10,000	www.tjgreenbio.com
Ecomann, Shenzhen, China		P (3HB-co-4HB)	10,000	ecomannbruce.plasway.com
Metabolix, United States		P (3HB-co-4HB)	5000	IP sold to CJ, Korea
Biocycle, Brazil	*Bacillus* sp	PHB	100	www.fapesp.br
Nodax, other mixed PHAs	*Pseudomonas putida*	P (3HB-co-3HV) P (3HB-co-3HHx)	25,000	Kaneka, Japan, Kaneka/P&G
Genecis, Canada	Not Known	P(3HB-co-3HV)	Not Known	genecis.co
Terra Verdae Bioworks, Canada		PHA	Not Known	terrverdae.com

PHA, Polyhydroxyalkanoate.

HB, Hydroxyalkanoate.

HV, Hydroxyvalerate.

HHx, Hydroxyhexanoate.

## 10 Conclusion and future perspectives

For the microbial production of several value added bio-products, C1 sources are considered as the most promising and Next-generation carbon sources. Significant efforts have been made on the production of PHAs from C1 sources as it is eco-friendly, economical feasible and it has closed carbon cycle. Several strategies such as processes and metabolic engineering have been evolved to enhance the production efficiency of PHAs and their co-polymers in native autotrophs and methylotrophs. These strategies have extended the utilization of C1 sources for the production of PHAs. Companies like Mango materials and Newlight Technologies have already achieved the goal in producing PHAs on commercial scale from C1 carbon substrates (Joo 2008). The real motive of PHA scale up production is to collaborate with waste fermentation facilities or power plant as they emit GHGs at a substantial rate. However, these waste gases might not contain the actual composition of C1 gases, but a potential still available for the development of a bioreactor. Thus, PHA production from C1 sources can be considered as the most effective solution for the generation of plastics (Joo 2008).

Several waste substrates have been exploited for the production of PHA. However, C_1_ compounds are a promising feedstock for the biotechnological production of value-added chemicals. These compounds would help protect dwindling fossil fuel resources, reduce greenhouse gas emissions, and contribute to a cleaner climate. In the present scenario, it is easy to modify the microorganisms that contain the indigenous substrate pathways, for example, fixation of the CO_2_, degradation pathway, and CH_4_ oxidation. However, it is necessary to evaluate the theoretical limitations of the pathway. In this review, PHB production has studied from CO_2_, but the yield have been relatively low as compared to other carbon sources. To address this issue, several strategies have been developed to enhance the yield with better physicochemical properties. C1- fermenting microbes need to be engineered for the production of PHB with high yield and to tolerate other toxic gases. In addition to this, metabolic engineering approaches should be developed for the production of PHA copolymers. Life cycle assessment of C1- fermentation based biopolymer production needs to access the potential of C 1 sources mitigation and the circular economy. Furthermore, to evaluate the economic feasibility technoeconomic analysis should be done. All the discussed parameters need to be studied to replace synthetic plastics over biopolymers.

To overcome such problems, genetic engineering can be an approach. Optimizing the discovery and design of novel enzymes and pathways, modifying microorganisms, and developing innovative and efficient operating technologies will contribute towards the ultimate success of sustainable biopolymer production by utilizing C_1_ carbon sources. Synthetic biology and morphology engineering approaches will enhance the cell density and high volumetric productivity for process economics and suitable downstream development. To improve the thermal and mechanical properties of PHAs microorganisms are capable of utilizing various precursors through metabolic engineering approaches. Apart from this artificial intelligence helps to improve the industrial production of PHAs. To make PHAs production more economy, a co-production of PHAs with other chemicals such as ectoine, inositol, amino acids, hydroxy acids can be done. Next-Generation Industrial Biotechnology offers several opportunities for bioproduction of PHAs with fully automatic bioprocessing plants can be expected.
